# Funerals

**Published:** 1877-07

**Authors:** 


### FUNERALS.

Not an enticing theme for perusal, certainly, but one, nevertheless, that will enlist the attention of the thoughtful.

Funerals are a prolific source from which contagion spreads to depopulate a neighborhood, or a village, even, of its children! People who would shun the funeral of a child dead from small-pox, rush with alacrity to the death-bed of one that has perished from scarlet fever or diphtheria, never dreaming that small-pox is the least dangerous of the diseases mentioned, and none the more communicable by means of the clothing worn upon the infected premises. Small-pox is rendered comparatively harmless through vaccination, while diphtheria, scarlet fever and measles are spread among our innocent children by thoughtless persons with a penchant for funerals.

Be it known that, the diseases mentioned are highly contagious, and many times more dangerous in their consequences than the much dreaded small-pox; that they can be communicated from house to house in the clothing of visitors; that they are often sent hundreds of miles by means of a letter written in an infected house, and innocent households so made to suffer from the thoughtlessness of friends.

We hope our legislators will be aroused to the necessities of the case, and render it unlawful to have public funerals of persons who have died of diphtheria, scarlet fever, measles, or typhoid-fever, and compelling households so afflicted to display some signal, warning visitors from the premises. Until this is done, we may expect these highly contagious diseases to continue to destroy our children, almost to their entire extermination, as has occurred in numerous places within a few months.

